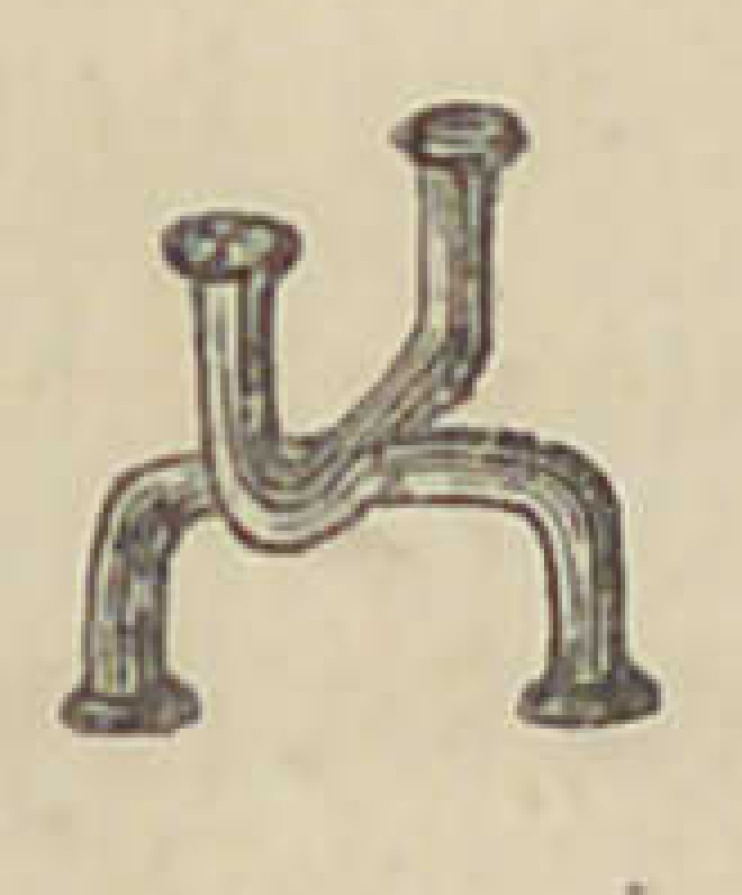# Improvement in Plate Teeth

**Published:** 1868-01

**Authors:** 


### IMPROVEMENT IN PLATE TEETH.

THE accompanying illustration presents an improvement in the manner of arranging the platinum pins in plate teeth, invented by Dr. JOHN A. MASON, of Keokuk, Iowa. The advantages of this arrangement will be quite apparent to any one at first view. Pins placed transversely in any single teeth weaken them very much; but especially in narrow teeth is this a serious difficulty, one that Dr. Mason's method will overcome. We are not advised as to the Dr's. method of making these double staple pins. Their manufacture by hand would be a slow process, and not an easy one to accomplish by machinery. However, we will not trouble ourselves about that, for the mind that conceived the idea will plan for its execution. We trust these teeth, or rather teeth with these pins will be manufactured, and put into the market at once. By their use a difficulty that has been long recognized as a serious one will be nearly if not altogether overcome.

## Figures and Tables

**Figure f1:**
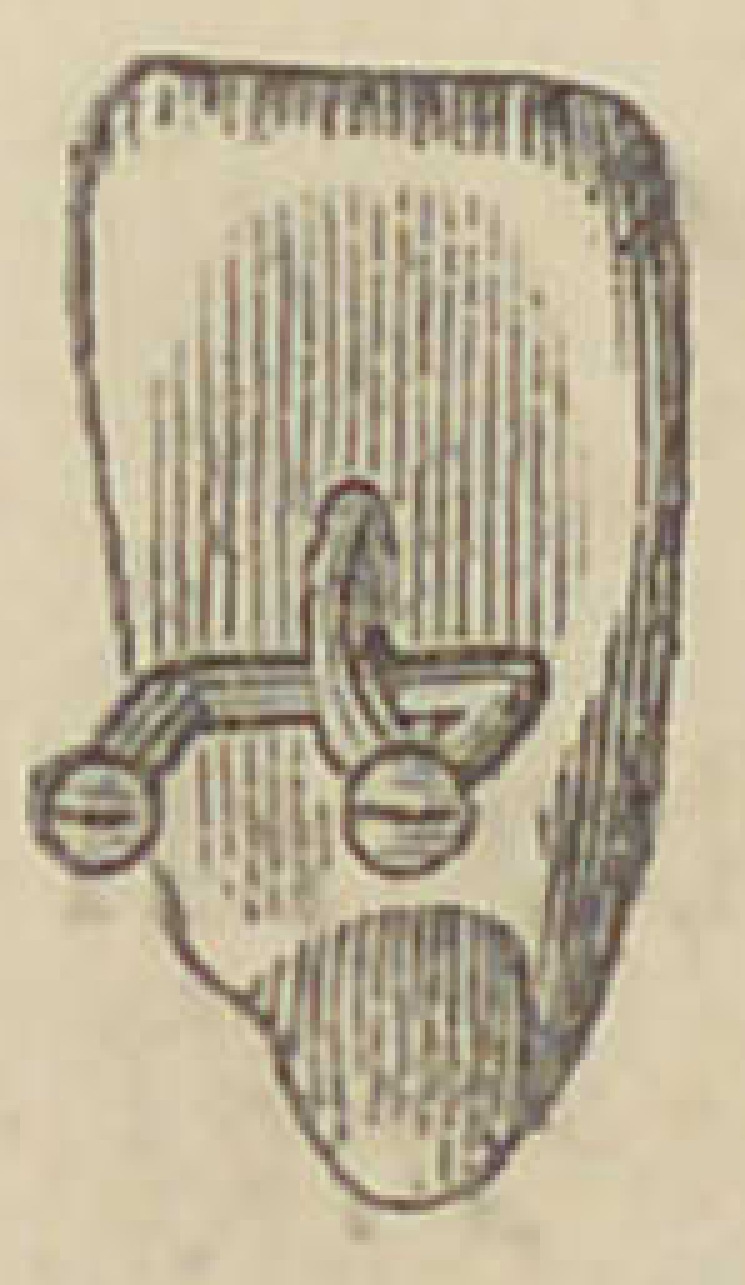


**Figure f2:**